# Peer-induced quiescence of male *Drosophila melanogaster* following copulation

**DOI:** 10.3389/fnbeh.2024.1414029

**Published:** 2024-07-16

**Authors:** Katrina Lynn, Toshiharu Ichinose, Hiromu Tanimoto

**Affiliations:** ^1^Graduate School of Life Sciences, Tohoku University, Sendai, Japan; ^2^Frontier Research Institute for Interdisciplinary Sciences, Tohoku University, Sendai, Japan

**Keywords:** post-copulatory behavior, *Drosophila melanogaster*, male behavior, peer-induced behavior, quiescence

## Abstract

Mating experience impacts the physiology and behavior of animals. Although mating effects of female *Drosophila melanogaster* have been studied extensively, the behavioral changes of males following copulation have not been fully understood. In this study, we characterized the mating-dependent behavioral changes of male flies, especially focusing on fly-to-fly interaction, and their dependence on rearing conditions. Our data demonstrate that male flies quiesce their courtship toward both females and males, as well as their locomotor activity. This post-copulatory quiescence appears to be contingent upon the presence of a peer, as minimal variation is noted in locomotion when the male is measured in isolation. Interestingly, copulated males influence a paired male without successful copulation to reduce his locomotion. Our findings point to a conditional behavioral quiescence following copulation, influenced by the presence of other flies.

## Introduction

Across diverse animal taxa, mating is a pivotal event that is crucial for the passage of genes and has been a focal point of scientific inquiry for many years. Premating physiological changes and behavioral patterns, such as estrus and courtship displays, have garnered extensive research attention (Bastock and Manning, 1955; Erskine, 1989; Anholt et al., 2020). Mating can induce significant behavioral modifications (Cury et al., 2019; Anholt et al., 2020), and post-mating changes play a crucial role in individual fitness. Seminal fluid, given to the female through copulation, not only facilitates the up-regulation of antimicrobial genes, but also influences fecundity and sperm storage dynamics in insects (Avila et al., 2011; Perry et al., 2013). Considering that many animals mate multiple times throughout their lives, and the potential consequences of each copulatory event, a thorough examination of post-copulatory behaviors becomes imperative. Extensive research into female *Drosophila melanogaster* has unveiled notable post-copulatory phenomena, including an increase in egg laying (Soller et al., 1999) and feeding (Carvalho et al., 2006), and an overall decline in receptivity toward subsequent mating events (Manning, 1967).

An aspect known to influence sexual behaviors in *Drosophila melanogaster* is the rearing condition of the fly (Chen and Sokolowski, 2022). Goncharova et al. (2016) demonstrated that flies raised in social groups exhibit an overall reduction in locomotor activity, along with a decrease in the production of the male courtship song toward females. Furthermore, Marie-Orleach et al. (2019) revealed that social isolation shortens bursts of male courtship song, while Sethi et al. (2019) showed that isolation can diminish the overall success rate of courting males. Additionally, Kim et al. (2012) noted that males raised among rival males significantly increase their copulation duration. These collective findings highlight the influence of rearing conditions, such as group rearing and social isolation, on a wide spectrum of sexual behaviors. However, their specific impact on post-copulatory behavior remains to be elucidated.

The bulk of research focusing on male sexual behavior has predominantly concentrated on the examination of innate pre-copulatory behaviors, as comprehensively reviewed by Yamamoto et al. (1997). However, a limited number of studies have explored post-copulatory behavior, often focusing instead on unraveling the neural circuitry associated with post-copulatory sexual satiation and subsequent reduction in courtship behavior. One such study by Zhang et al. (2016) endeavored to elucidate the impact of dopaminergic circuitry on male post-mating sexual drive. Furthermore, ongoing research aims to unravel the intricacies underlying the post-copulatory reduction in courtship behavior, with studies positing a courtship circuit mechanism under homeostatic control (Zhang et al., 2019; Jung et al., 2020). Despite these notable contributions, research into the post-copulatory behavior of male flies remains relatively limited within scientific literature.

Considering that the reduction of sexual motivation following copulation is already known (Manning, 1967), we aimed to comprehensively characterize any and all behavioral changes of male flies after copulation and to elucidate the impact of rearing condition on these changes.

## Materials and methods

### Flies

The wild-type *Drosophila melanogaster* Canton-S (CS) strain was utilized in this study. Flies were reared and maintained on standard cornmeal food at 24°C under a 12–12 h light–dark cycle for all experiments. We used 3–4 day old flies for all experiments.

### Animal preparation

#### Rearing condition following eclosion

We prepared single housed and group housed flies in a similar manner to previous studies (Pan and Baker, 2014; Kohatsu and Yamamoto, 2015; Bentzur et al., 2021). Single housed males were isolated directly following eclosion and for 3–4 days post-eclosion, while group housed males were kept in monosexual groups of 10 for the same period.

#### Sexual experience before behavioral measurement

To investigate the behavioral effect of copulation, we prepared four distinct groups of flies: “successful,” “rejected,” “friendzoned,” and “naïve,” each representing different sexual experiences, and subjected them to a two-hour period of pre-measurement preparation in thin plastic food vials. “Successful” males were provided with 5 virgin females and checked to confirm whether they had mated at least once in the two hours. Males that did not engage in copulation during this period (~5% of all males) were excluded from subsequent analysis.

Females who have copulated are known to reject males’ courtship attempts (Connolly and Cook, 1973), so for the “rejected” group, males were paired with a single mated female for a two-hour duration. Mating of the target female occurred 1 day prior to the behavioral measurement. A minority of males managed to copulate with the mated female and were excluded from the further analysis.

In the “friendzoned” group, males were paired with a single virgin female whose vaginal plates had been sealed with an ultraviolet-cured adhesive [Norland Optical Adhesive 68 (Norland Products Inc., Jamesburg, NJ. P/N 6801)], a procedure conducted one hour prior to the pairing using cold-anesthetization. This novel setup afforded males an opportunity to interact with living virgin females without the possibility of copulation or rejection.

“Naive” males were kept in isolation for the duration of the for two hour preparation period.

#### Behavioral measurements

For paired male measurements, two males were gently aspirated into arenas with a diameter of 30 mm and a height 8 mm. Prior to pairing, the flies were allowed to acclimate for 15 min individually via separation by a filter paper barrier. Following this acclimation period, the flies were paired by gently removing the filter paper, resulting in a single arena. A square coverglass lid (Matsunami Micro Cover Glass, 30×30 mm, thickness 0.13 ~ 0.17 mm, Osaka, Japan P/N: C030301) coated with Sigmacote (Sigma-Aldrich, Tokyo, Japan P/N: SL2). was used to cover the arena. The inner walls of the arenas were coated with Fluon (Insect-a-Slip PTFE30, BioQuip Products, Rancho Dominquez, CA P/N: #2871C) to prevent the flies from climbing. The prepared arena was placed on a glass surface and illuminated from below using an LED light box (400-TBL003, SANWA supply). Behaviors were video recorded using a camera positioned approximately 60 cm above the arena, with a frame rate of 30 frames per second (Pentax Q-S1, Pentax).

For male–female pairs, behaviors were video recorded for up to 5 min before copulation, approximately 20 min during copulation, and 25 min after copulation. For male–male pairs, behaviors were recorded continuously for 25 min after copulation.

### Behavioral classification and measurement

#### Courtship index

The Courtship Index (CI) is calculated as the duration of time a male spends engaged in courtship behaviors, divided by the total duration of the recording. Stereotypical courtship behaviors include approach, genital licking, unilateral wing extensions, and attempted mounting. These behaviors were annotated using BORIS [Behavioral Observation Research Interactive Software (Friard and Gamba, 2016)]. To evaluate post-copulatory sexual behavior, the CI was measured for a period of 5 min following unmounting, which marked the end of copulation. Pre-mating CI was assessed by measuring courtship activities until copulation occurred, for a duration of up to 5 min.

#### Locomotor activity and inter-individual distance

The positions of male flies and their linear velocity were quantified using the fly tracking software TPro (Sirigrivatanawong et al., 2017; Okuno et al., 2019) for a total time of 25 min after copulation. A correlation plot between inter-individual distance and velocity was calculated based on the two-dimensional kernel density estimation using *R*.

### Statistical analysis

We checked for normality using the Jarque-Bera test. If the data did not violate the assumption of normality, we conducted a one-way ANOVA with an independent samples *t*-test unless stated otherwise, paired samples *t*-test). In cases where the data were not normally distributed, we employed the Kruskall-Wallis test for variance followed by the Mann–Whitney U test. All statistical tests were corrected using the Benjamini Hochberg correction. The significance level for all statistical analyses was set to 0.05 (two-sided test). All statistical analyses were conducted using Microsoft Excel.

## Results

### Mating reduces sexual motivation and locomotor activity

**Figure 1:**
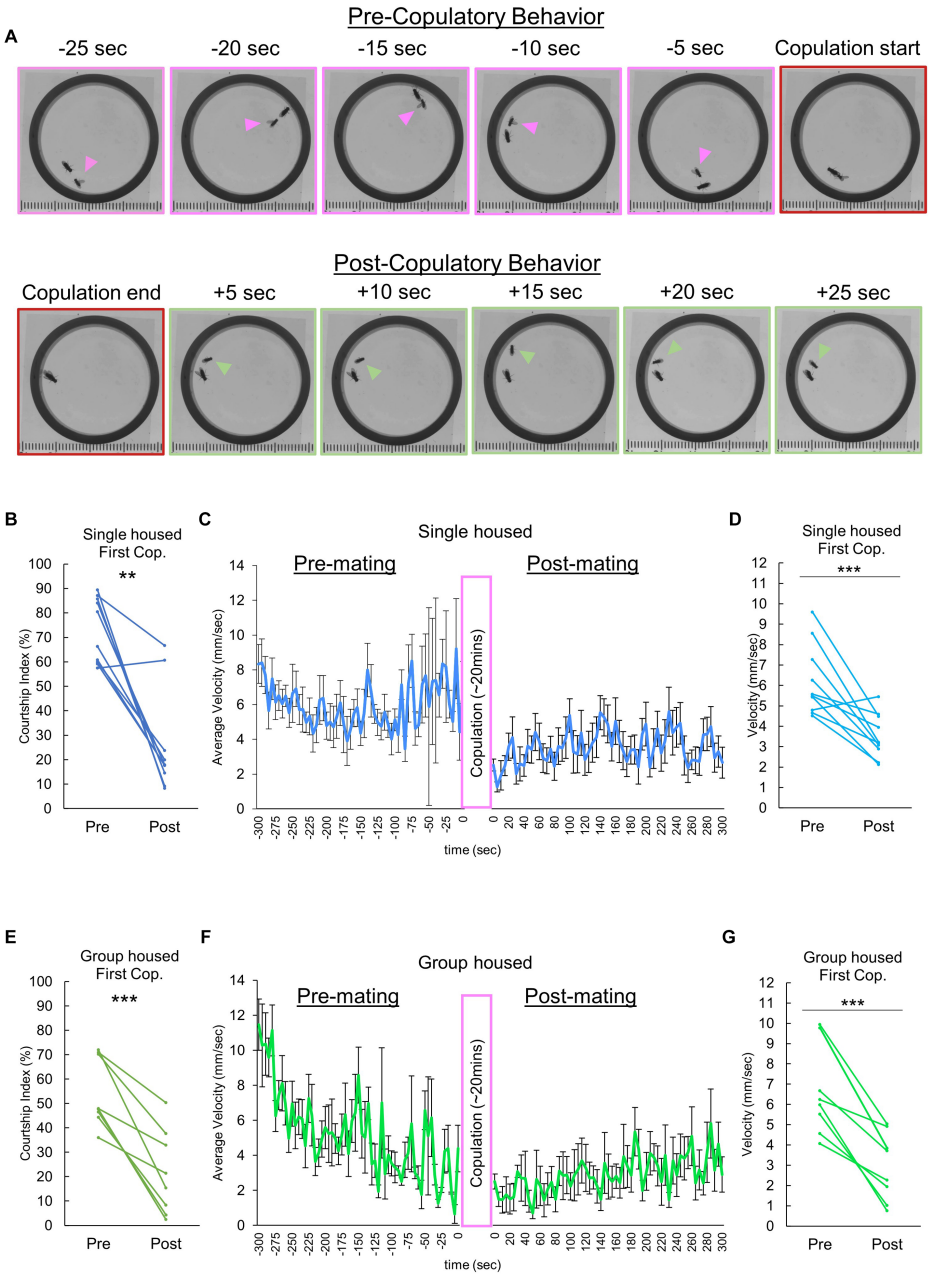
Decreases in male sexual motivation and velocity following copulation. **(A)** Video frames illustrate the behavioral dynamics of single-housed males following copulation. Arrowheads denote the male. Frames with distinct pink and green borders represent instances of courtship behavior and grooming/resting, respectively. **(B)** Changes in courtship behavior, quantified by the Courtship index (CI), are observed in single housed males before and after copulation with a conspecific female. Multiple pair-wise comparisons are made using paired samples *t*-tests followed by the Benjamini-Hochberg correction, with significance levels indicated by asterisks (**q < 0.01). Sample size, *n* = 10. **(C)** Temporal locomotor patterns of individually reared male subjects before and after mating. The average velocity for all male subjects is plotted. Sample size *n* = 10. **(D)** Average locomotor velocity observed in individually reared males before and after copulation with a conspecific female. Statistical analysis is performed using multiple pair-wise comparisons made by paired samples *t*-tests followed by the Benjamini-Hochberg correction. ***q < 0.001, *n* = 10. **(E)** Changes in courtship behavior, quantified by the Courtship index (CI), are observed in group housed males before and after copulation with a conspecific female. Multiple pair-wise comparisons are conducted using paired samples *t*-tests followed by the Benjamini-Hochberg correction, with significance levels indicated by asterisks (***q < 0.001). Sample size *n* = 8. **(F)** Temporal locomotor patterns of group housed male subjects before and after mating. The average velocity of all male subjects is plotted. Sample size *n* = 8. **(G)** Differences in velocity observed in group housed males before and after copulation with a conspecific female. Multiple pair-wise comparisons are made using paired samples *t*-tests followed by the Benjamini-Hochberg correction, with significance levels indicated by asterisks (***q < 0.001) and sample size *n* = 8.

To explore potential mating-dependent behavioral changes, we video recorded and compared males in the pre- and post-copulatory phases of mating. There was a shift in behavior following copulation (Figure 1A), notably a reduction in courtship behavior directed toward the female. To quantify this, we analyzed the courtship index of the male fly, which revealed a sharp decline in courtship activity after their initial mating experience (Figure 1B). Subsequent quantitative analyses of average overall velocity and individual male velocity differences pre- and post-copulation confirmed a significant reduction in male flies’ overall movement (Figures 1C,D). Further investigation aimed to characterize how a males’ previous housing experience could influence the observed behavioral quiescence. Our initial data was comprised of males raised 1 fly per vial for 3–4 days directly following eclosion. Additionally, we evaluated group reared males (“group housed” with 10 male flies per vial, raised 3–4 days from eclosion). Akin to individually raised males, group housed males exhibited a significant decrease in courtship index (Figure 1E), and a marked reduction in velocity following copulation (Figures 1F,G).

While Figure 1 primarily addressed behavioral changes following a single copulation, it is important to acknowledge that most *Drosophila* males will mate multiple times when given the opportunity. Thus, we extended our investigation to examine the observed quiescent behavior following repeated copulations.

**Figure 2:**
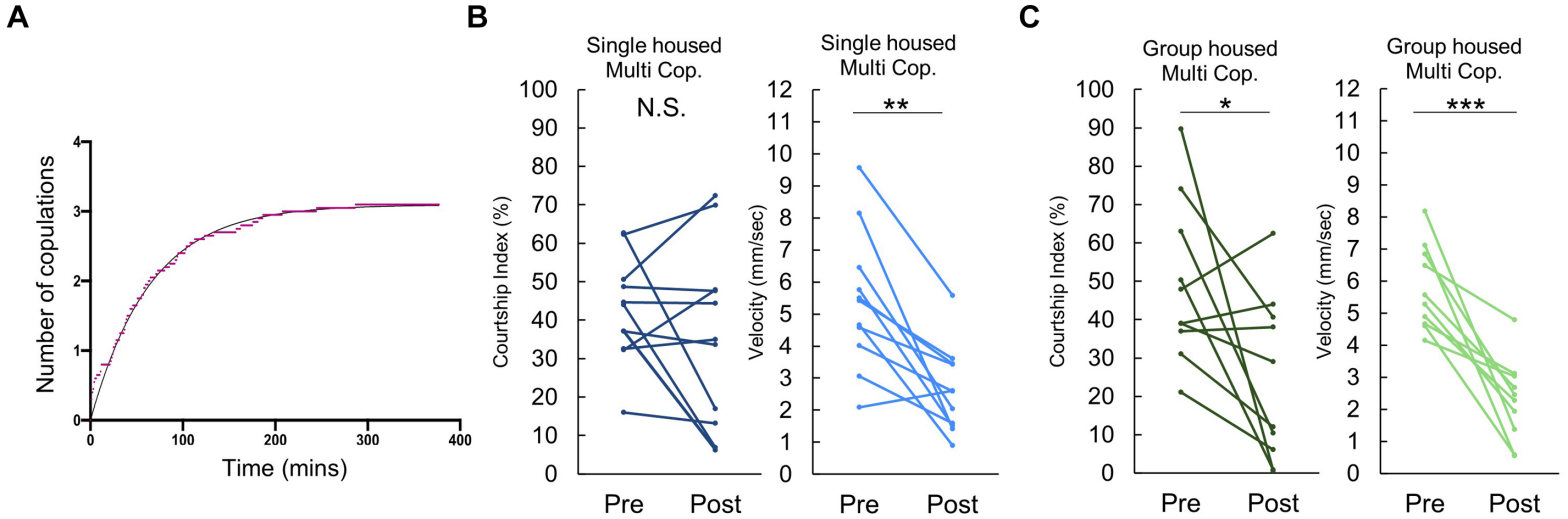
Males conditionally decrease their courtship activity following multiple copulations. **(A)** Cumulative number of copulations observed in individually reared males when paired with 5 virgin females. The x- and the y- axes represent time and the number of copulations, respectively. **(B)** Changes in Courtship Index (CI) and locomotor activity, as measured by average velocity, in individually reared males before and after three copulatory events with a virgin female. Multiple pair-wise comparisons are made using paired samples *t*-tests followed by the Benjamini-Hochberg correction. Significance levels indicated by asterisks: *q < 0.05, **q < 0.01, ***q < 0.001, *n* = 11. **(C)** Changes in Courtship Index (CI) and locomotor activity, as measured by average velocity, in group housed males before and after three copulatory events with a virgin female. Multiple pair-wise comparisons were made by paired samples *t*-tests followed by Benjamini-Hochberg correction. *:q < 0.05, **:q < 0.01, ***:q < 0.001. n = 10 (Group housed).

When presented with multiple females, a majority of males mated approximately 3 times before reaching a plateau of “sexual satiety” (Figure 2A). Given this, we opted to examine behavior following 3 successive copulations. Intriguingly, after the third mating, single housed males exhibited no significant decrease in post-copulatory courtship behavior (Figure 2B), despite experiencing a decline in velocity similar to that observed following their first copulation. In contrast, group housed males displayed a reduction in both courtship index and velocity (Figure 2C). This difference may stem from the observation in Figure 2C, that pre-third mating, single housed males already exhibit a considerably lower Courtship Index than their group housed counterparts. These findings suggest that mating induced courtship reduction depends on previous sexual experience and rearing condition.

**Figure 3:**
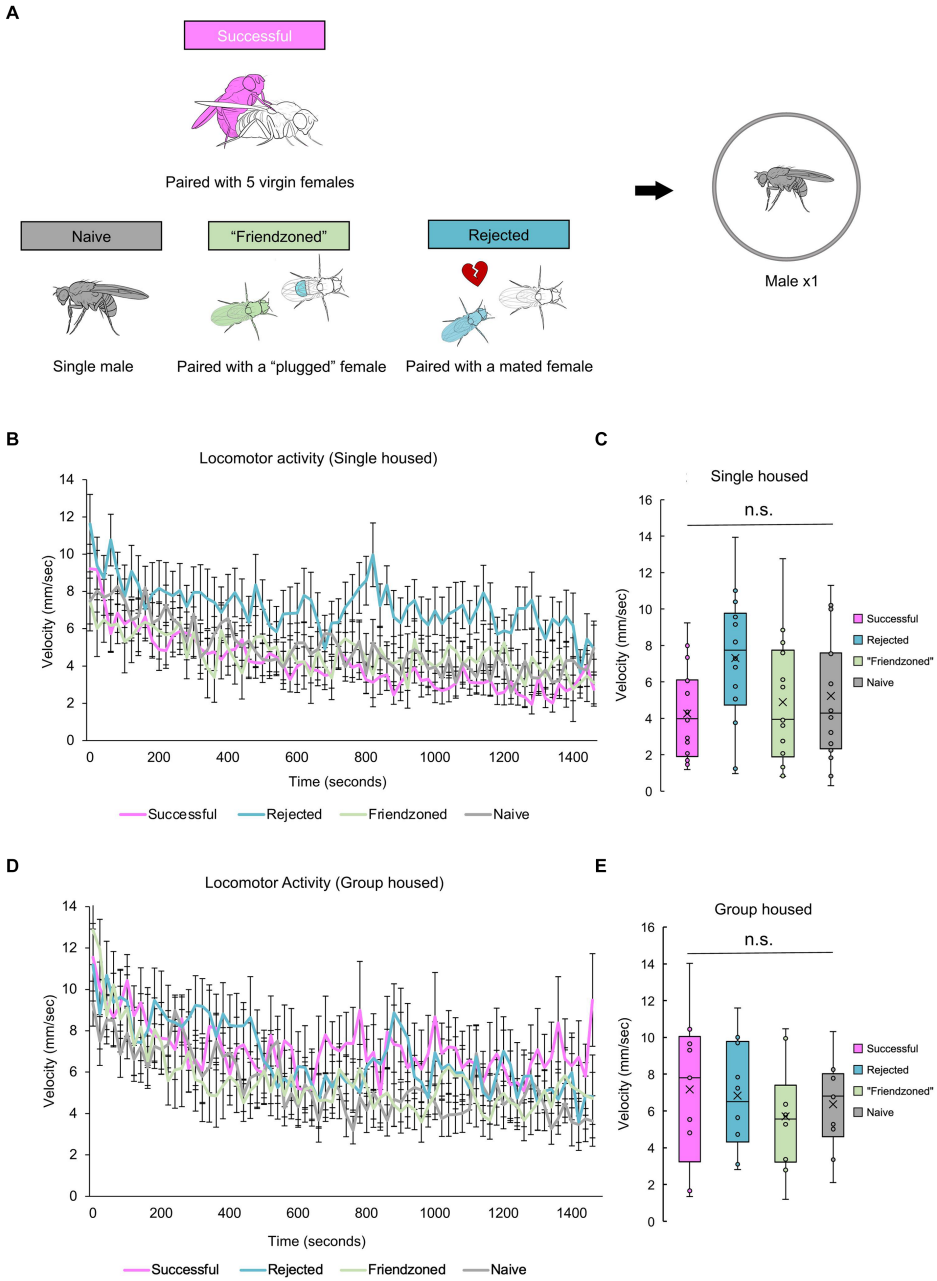
Successful males do not decrease locomotor activity when measured alone. **(A)** Description of control groups and the experimental procedure. Male flies are placed in food vials for a 2-h period, accompanied by different social stimuli: 5 virgin females (Successful), a mated female (Rejected), a virgin female whose genitalia was covered (“Friendzoned”), or in the absence of any flies (Naive). Subsequently, individual flies are transferred to observation arenas for a 25-min video recording. **(B)** Temporal profile of average locomotor activity of singly reared males over the entire 25-min measurement. The error bars represent the standard error of mean (s.e.m.). **(C)** Locomotor activity averaged in the whole measurement. Statistical analysis is conducted using one-way ANOVA, with non-significant differences indicated as n.s.: *q* > 0.05. Sample sizes: *n* = 16 (Successful); 14 (Rejected); 14 (Friendzoned); 16 (Naive). **(D)** Temporal profile of average locomotor activity of group reared males over the entire 25-min measurement. The error bars represent the standard error of mean (s.e.m.). **(E)** Locomotor activity averaged in the whole measurement. Statistical analysis is conducted using one-way ANOVA, with non-significant differences indicates as n.s.: *q* > 0.05. Sample sizes, *n* = 9 (Successful); 10 (Rejected); 10 (Friendzoned); 10 (Naive).

So far, post-copulatory behavioral quiescence, characterized by a consistent decrease in locomotor activity and occasionally a decrease in mating behavior following copulation, has been observed when a male was paired with a female. This observation has led to the hypothesis that the reduction in courtship behavior following copulation could be attributed to sexual demotivation. Alternatively, we considered the possibility that copulation might induce a broader quiescence of behavior. To test this hypothesis, we investigated whether a male fly would exhibit quiescence following copulation when alone, under various experimental conditions. We chose to further examine the behavior of “sexually satiated” males and introduced three control groups for comparative analysis. These control groups underwent a 2-h “conditioning” period and included the “rejected” male group, who was paired with a mated female that actively rejected him; the “friendzoned” group, who was paired with a virgin female whose genitalia were covered to prevent copulation; and the “naive” group, who was isolated before the locomotion measurement and treated as a pre-copulation control (Figure 3A). These males were then tested directly following the 2 h sexual experience. Interestingly, “successful” males displayed walking speeds indistinguishable from those of control males over the long-term, regardless of the rearing conditions (Figures 3B–E). These results suggest that sexual demotivation may underlie the post-copulatory quiescence, and that isolation appears to obscure the previously observed behavior (Figures 1, 2).

### Post-copulatory quiescence in paired “successful” males

**Figure 4:**
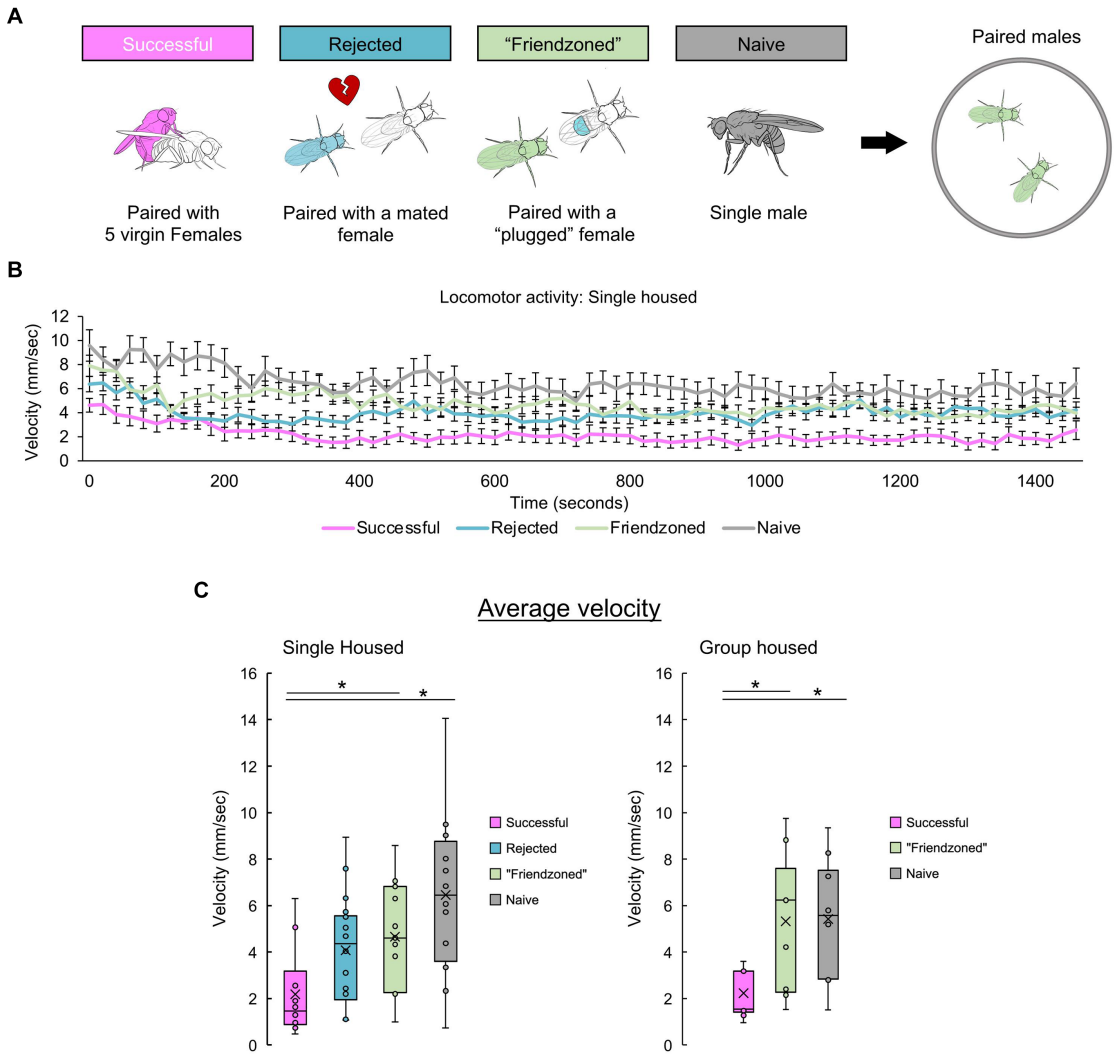
Successful males quiesce when paired with another male. **(A)** Experimental conditions of male *Drosophila melanogaster* to investigate the influence of sexual experiences and rearing conditions on post-mating behaviors. Behavioral measurements are conducted in paired male flies with the same past experiences. **(B)** Temporal profile of post-mating locomotion in paired males’ compared across different control groups. The average velocity of two flies is plotted. **(C)** Mean velocity over the entire 25-min recording period, presented separately for both single housed (left) and group housed (right) males. For comparisons between single housed (SH) groups, a one-way ANOVA followed by independent-samples *t*-test with Benjamini-Hochberg correction for multiple pairwise comparisons is performed. For comparisons between group housed (GH) groups, the Kruskall-Wallis test followed by Mann–Whitney U test with Benjamini-Hochberg correction for multiple pairwise comparisons are performed. Significance is denoted by asterisks (*q < 0.05). Sample sizes are *n* = 10 (Successful), 18 (Rejected), 15 (Friendzoned), 12 (Naive) for single housed and *n* = 11 (Successful), 9 (Friendzoned), 10 (Naive) for group housed.

**Figure 5:**
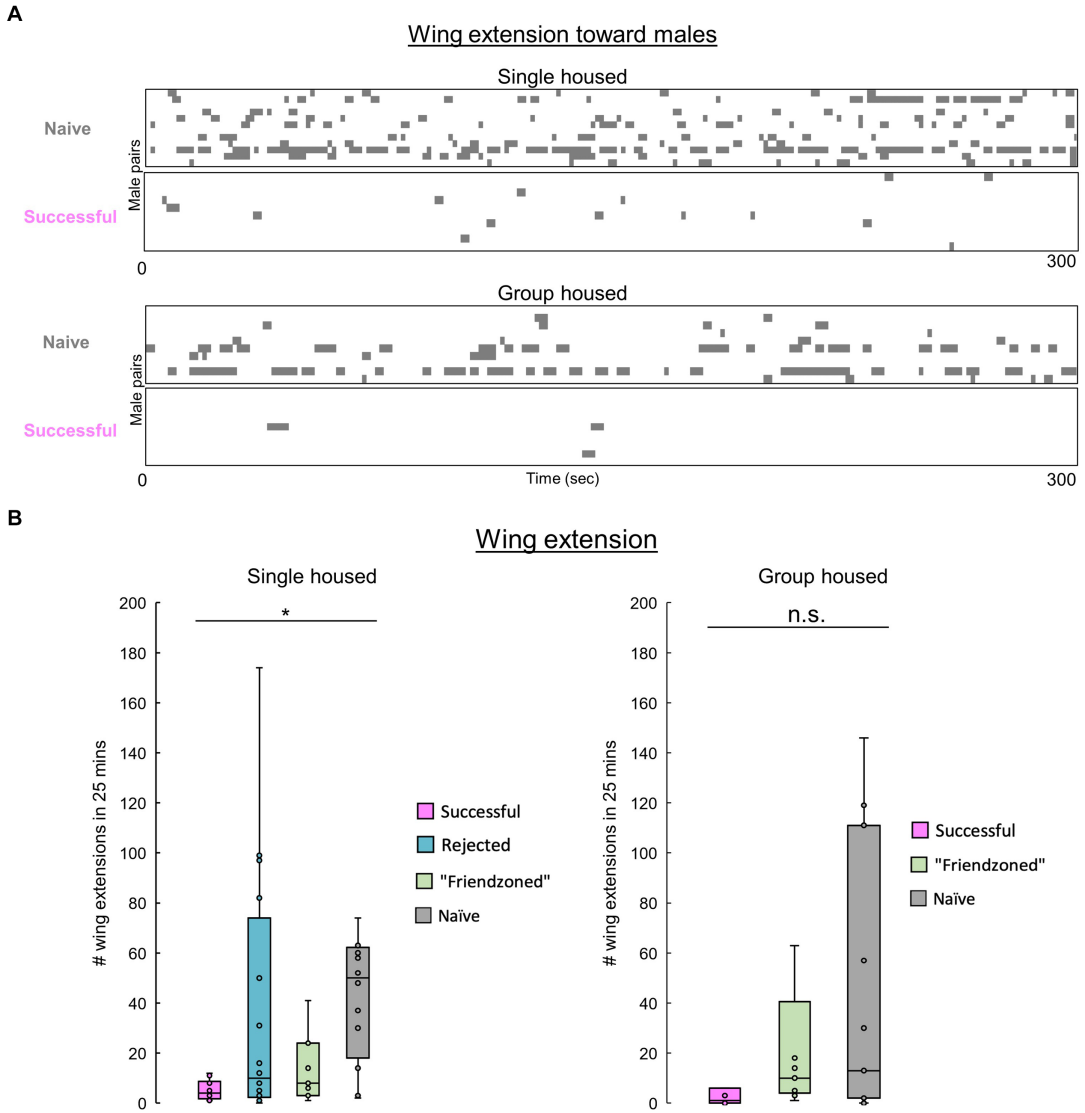
Males decrease their homosexual courtship following copulation. **(A)** Raster plots of unilateral wing extensions in paired successful or naïve males during the first 5 min of pairing. **(B)** Total number of wing extensions among males over the entire 25-min testing period. The Kruskall-Wallis test followed by the Mann–Whitney U test with Benjamini-Hochberg correction for multiple pairwise comparisons is performed. Significance is denoted by asterisks (*q < 0.05), and sample sizes are *n* = 10 (Successful), 9 (Friendzoned), 11 (Naive) for single housed; Kruskall-Wallis *p* > 0.05, *n* = 11 (Successful); 16 (Rejected); 11 and (Friendzoned); 12 (Naive) for group housed.

To delve deeper into the behavioral modifications triggered by mating in male flies and explore their reliance on the presence of conspecifics, we paired “successful” males and assessed them alongside control pairs (Figure 4A). Given the consistent similarity in results between the friendzoned and rejected groups, we opted to use the friendzoned group as the only group housed control from Figure 4 through Figure 5. This group’s pairing with a virgin female renders it the optimal control. Remarkably, “successful” pairs demonstrated a marked decrease in velocity during the 25-min observation period, consistent with the decline observed in Figure 1 (Figure 4B). Importantly, this reduction occurred consistently in both individually housed and group housed males. Together with the results in Figure 3, we have done a two-way ANOVA, with mating experiences and the testing conditions as the two factors and found a significant interaction (SH: *p* = 0.027, GH: *p* = 0.035). Combined with the results of Figure 3, we conclude that post-copulatory quiescence is dependent on conspecific presence.

**Figure 6:**
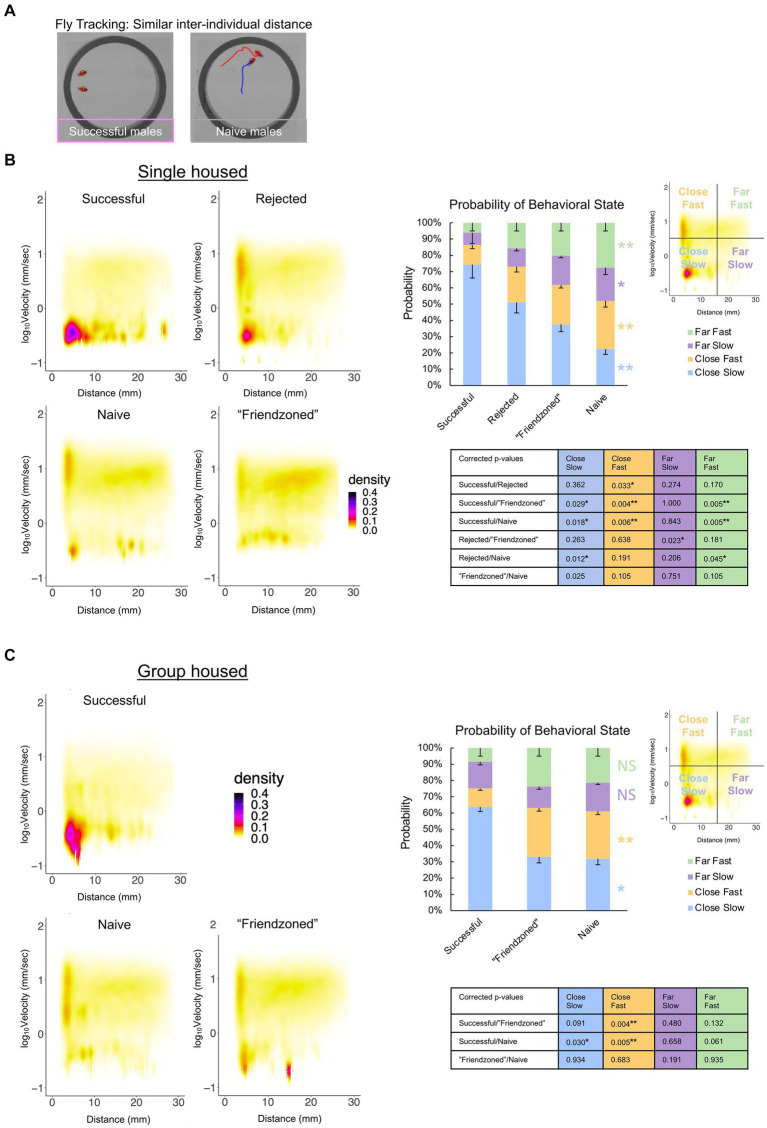
Sexual experience affects behavioral dynamics of paired male flies. **(A)** Locomotor traces of successful and naive males from the single housed (SH) condition. The red and blue lines indicate the trajectories of the two individuals (red circled). **(B)** Two-dimensional kernel density estimation plot of velocity and inter-individual distance of the two flies in the single housed condition, indicated with pseudo-color. The percentage of values per quadrant, dependent on the experimental condition, is shown on the right. The Kruskall-Wallis test is performed, and significance is displayed to the right of the bars. The Mann Whitney U test with Benjamini-Hochberg correction for multiple pairwise comparisons is subsequently performed with results shown in the *p*-value table. (*q < 0.05, **q < 0.01). Sample sizes are *n* = 10 (Successful), 18 (Rejected), 15 (Friendzoned), 12 (Naive). **(C)** Two-dimensional kernel density estimation plot of velocity and inter-individual distance of the two flies from the group housed (GH) condition, indicated with pseudo-color. The percentage of values per quadrant, dependent on the experimental condition, is displayed on the right. The Kruskall-Wallis test is performed, and significance is displayed to the right of the bars. The Mann Whitney U test with Benjamini-Hochberg correction for multiple pairwise comparisons is subsequently performed and results are displayed in the *p*-value table. (*q < 0.05, **q < 0.01), and sample sizes are *n* = 11 (Successful), 9 (Friendzoned), 10 (Naive).

Additionally, we observed a spectrum of distinct behavioral phenotypes among paired males, ranging from sitting in close proximity to chasing and courting one another (Figure 6A), leading us to hypothesize that these various behavioral states may be contingent on the males’ prior sexual experiences. To better examine and define the behavioral phenotypes occurring, we conducted a comprehensive analysis that considered both inter-individual distance and velocity concurrently. From this analysis, we observed that “successful” male pairs displayed a significantly higher probability of low velocity coupled with short inter-individual distances compared to the naive and other controls, indicative of peer-induced post-coital quiescence. Conversely, control males exhibited higher velocity levels at short inter-individual distances, suggestive of chasing behavior (Figures 6B,C).

Previous data indicated that naive flies exhibit heightened courtship behavior, typically characterized by increased velocity, and reduced inter-individual distance. To confirm this, we measured unilateral wing extension across conditions. Notably, “successful” males, despite no statistical significance in the group housed conditions, consistently exhibited a decreased level of courtship behavior compared to other groups (Figures 5A,B).

### Mating experience induces quiescence in an associated “rejected” male

**Figure 7:**
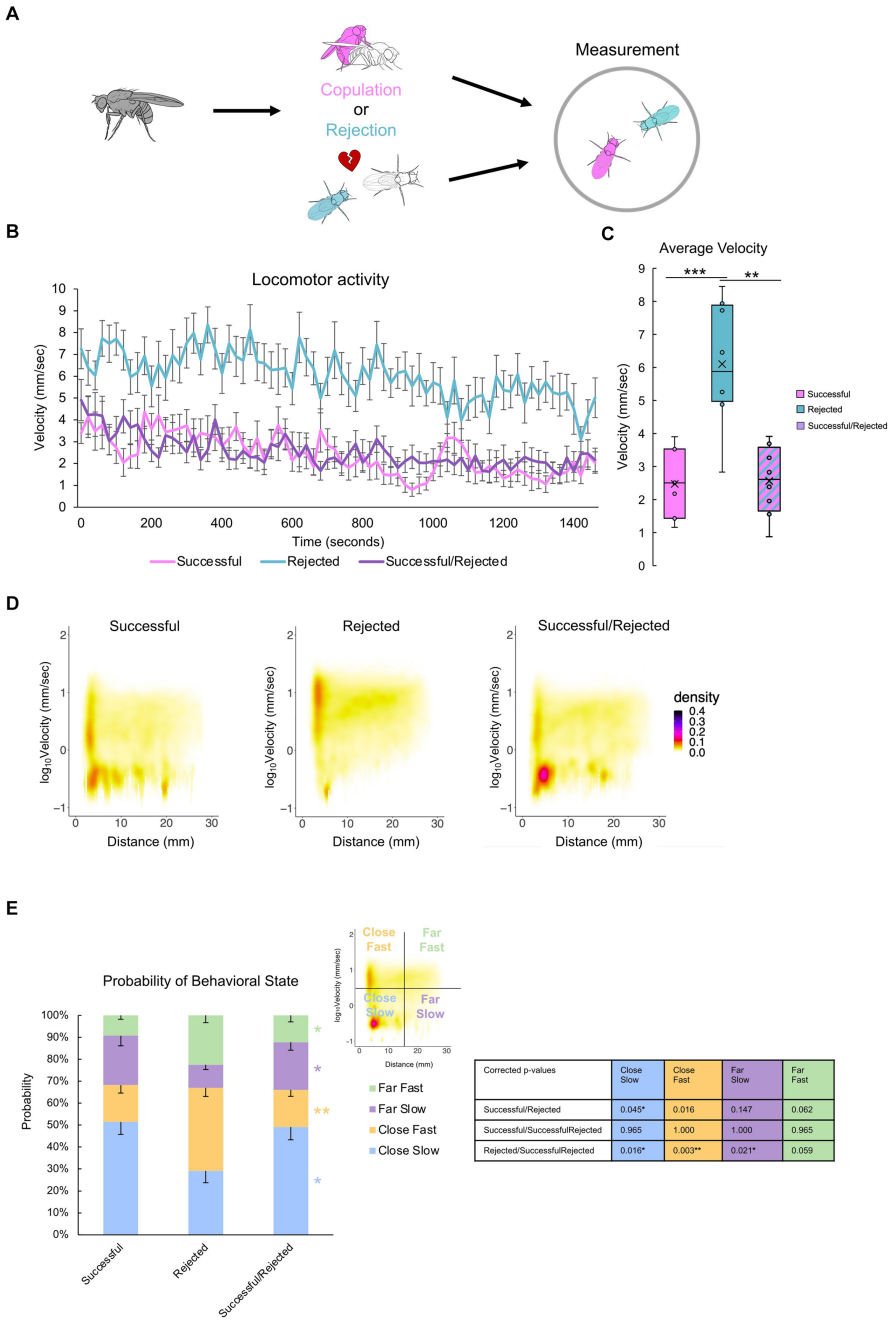
Mating experience induces quiescence of a paired “rejected” male.**(A)**Description of the control groups and experimental procedure. Males with different sexual experiences are paired for two hours. **(B)** Time course of locomotion over the entire 25-min measurement. Note that singly reared males are measured. **(C)** Average velocity of each group. One-way ANOVA, and independent-samples t-test with Benjamini-Hochberg correction are performed, with significance denoted with asterisks (*q < 0.05, **q < 0.01, ***q < 0.001), and sample sizes *n* = 7 (Successful); 8 (Rejected); 8 (Successful/Rejected). **(D)**Two-dimensional kernel density estimation plot of velocity and inter-individual distance of the two flies, indicated with pseudo-color. **(E)** The percentage of values per quadrant, dependent on the experimental condition, is shown on the right. The Kruskall-Wallis test is performed, and significance is displayed to the right of the bars. The Mann–Whitney U test with Benjamini-Hochberg correction for multiple pairwise comparisons is subsequently performed, with results shown in the *p*-value table. *p* < 0.001, *n* = 7 (Successful); 8 (Rejected); 8 (Successful/Rejected).

Previous experiments demonstrated the critical role of a paired fly in the post-coital behavioral quiescence of males (Figures 3, 4). However, the extent to which flies influence one another is unclear. To address this, we investigated the impact of one male’s sexual history on another male (Figure 7A). Pairing a “rejected” male with a “successful” male revealed that the average velocity decreased to levels comparable to those observed in pairs of “successful” males, and markedly lower than in pairs of “rejected” males (Figures 7B,C). The hetero-paired males maintained close proximity to each other while displaying a pronounced reduction in velocity (Figures 7D,E), reminiscent of the behavioral pattern observed in “successful” male pairs (Figure 6). This observation suggests that the mating experience of the “successful” male can induce quiescence in the “rejected” male, emphasizing the influential nature of mating-induced behavior among male flies.

## Discussion

Our findings reveal that male *Drosophila melanogaster* exhibit reduced courtship behavior toward both females (Figures 1, 2) and other males (Figure 5) following copulation, accompanied by a decrease in overall velocity. These results, in conjunction with the findings that males who are alone do not alter their velocity (Figure 3), suggest that post-coital quiescence manifests only in the presence of other flies, whereas it is absent when experienced males are alone. Moreover, our findings reveal the intriguing observation that “successful” males tend to position themselves in close proximity to each other without actively engaging in courtship behavior – an observation warranting further investigation. Furthermore, we can conclude from Figure 7 that the behavior of the rejected male can be influenced by the successful male. We can interpret this to mean that post-copulatory quiescence is likely due to the successful males’ lack of interaction, potentially influencing the rejected males’ activity.

Interestingly, in the single housed condition, we found particularly elevated locomotor activity and courtship behavior in the naive group compared to the others (Figures 4–6). In the group housed males, on the other hand, the naïve and the friendzoned exhibited comparable velocity and courtship behavior. This difference could potentially be attributed to their absence of exposure to other flies in the single housed naïve animals, which likely fosters increased peer interaction.

Post-coital quiescence is not unique to *Drosophila melanogaster* and is observed across various species. In mammals, male rats display diminished anxiety levels and reduced sexual motivation toward estrus females following copulation (Van Furth and Van Ree, 1996; Waldherr and Neumann, 2007). Likewise, in arthropods, there are intriguing examples of mating behaviors, such as those observed in males of the fishing spider species *Dolomedes tenebrosus*. These males exhibit a self-sacrificial behavior wherein they arrest their movement following copulation, ultimately allowing themselves to be consumed by the female as a source of nutrition (Schwartz et al., 2013).

With regard to physical changes that occur following copulation, Zhang et al. (2016) highlighted the emptying of the male’s ejaculatory bulb following copulation. This emptying could have an effect on the quiescence of the male’s behavior, perhaps due to a restoration of sperm within the ejaculatory bulb. Further exploration into these underlying mechanisms is imperative, especially given the conservation of post-copulatory quiescence across different species. The presence of these pacified behaviors in a diversity of species suggests a broader ecological benefit to quiescence following copulation.

Furthermore, our study underscores the significance of conspecific presence for males to exhibit behavioral quiescence. This observation aligns with the study by Zhang et al. (2016), which reported similar levels of locomotor activity post-copulation when flies were assessed individually. This group-induced behavior, reminiscent of the concept of “safety in numbers,” has been documented in various animals such as fish and butterflies (Finkbeiner et al., 2012; Johannesen et al., 2014). This suggests that sexually successful males may adopt conditional clustering behaviors for collective vigilance, potentially mitigating predation risk or conserving resources following mating. Intriguingly, our results indicate that this quiescence also influences males with varied past experiences (Figure 7).

Regarding potential neural correlates for this behavior, investigations into copulation-induced changes in neural activity have identified various factors involved, including serotonin (Norville et al., 2010). At the termination of copulation, the transfer of sperm from male to female is facilitated by an increase in serotonin levels (Tayler et al., 2012). This neurotransmitter has also been implicated in various aspects of sexual behavior, behavioral quiescence, social affinity, and social approach in *Drosophila* (Pooryasin and Fiala, 2015; Sun et al., 2020). The involvement of serotonin in sperm transfer suggests a plausible mechanism for the subsequent behavioral changes following copulation, as supported by our findings.

## Data availability statement

The original contributions presented in the study are included in the article/supplementary material, further inquiries can be directed to the corresponding author.

## Ethics statement

The manuscript presents research on animals that do not require ethical approval for their study.

## Author contributions

KL: Conceptualization, Data curation, Formal analysis, Investigation, Methodology, Writing – original draft, Writing – review & editing. TI: Conceptualization, Methodology, Supervision, Writing – original draft, Writing – review & editing. HT: Conceptualization, Funding acquisition, Methodology, Project administration, Resources, Supervision, Writing – original draft, Writing – review & editing.
